# Tracking the first SARS-CoV-2 Omicron BA.5.1.3 outbreak in China

**DOI:** 10.3389/fmicb.2023.1183633

**Published:** 2023-05-18

**Authors:** Xiaoxia Wang, Xiong Zhu, Yujin Lin, Lvfen He, Jing Yang, Chuan Wang, Wentao Zhu

**Affiliations:** ^1^West China School of Public Health and West China Fourth Hospital, Sichuan University, Chengdu, Sichuan, China; ^2^Central and Clinical Laboratory of Sanya People's Hospital, Sanya, Hainan, China; ^3^State Key Laboratory of Infectious Disease Prevention and Control, National Institute for Communicable Disease Control and Prevention, Chinese Center for Disease Control and Prevention, Beijing, China; ^4^Department of Infectious Diseases and Clinical Microbiology, Beijing Institute of Respiratory Medicine and Beijing Chao-Yang Hospital, Capital Medical University, Beijing, China

**Keywords:** SARS-CoV-2, Omicron BA.5.1.3, outbreak, mutation, Sanya

## Abstract

The SARS-CoV-2 is still undergoing rapid evolution, resulting in the emergence of several variants of concern, especially the Omicron variants (B.1.1.529), which are surging worldwide. In this study, we tracked Omicron subvariant BA.5.1.3 as the causative agent in the Hainan Province wave in China, which started on 1 August 2022. This was China’s first case of Omicron subvariant BA.5.1.3 and led to an indefinite total lockdown in Hainan with more than 8,500 confirmed cases. We obtained 391 whole genomes from positive nasopharyngeal swab samples in the city of Sanya in Hainan Province, which was the center of this outbreak. More than half of the infected cases were female (58%, 227/391) with a median age of 37.0 years (IQR 23.0–53.0). Median Ct values were 24.9 (IQR 22.6–27.3) and 25.2 (IQR 22.9–27.6) for *ORF1ab* and *N* genes, respectively. The total single-nucleotide polymorphism (SNP) numbers of Omicron BA.5.1.3 sampled in Sanya (median 69.0, IQR = 69.0–70.0) compared to those worldwide (median 63.0, IQR = 61.0–64.0) showed a significant difference (*p* < 0.05). Unique core mutations, including three non-synonymous mutations in *ORF1ab* (Y1064N, S2844G, and R3574K) and one synonymous mutation in *ORF3a* (S74S), were found. Phylogenetic analysis showed that virus from Sanya formed an independent sub-clade within the BA.5.1.3 subvariant, and could be divided into 15 haplotypes based on the *S* gene. The most recent common ancestor for the virus from Sanya was estimated as appearing on 5 July 2022, with 95% HPD ranging from 15 May to 20 September 2022. Thanks to our results, we were also able to delineate the mutational profile of this outbreak and highlight the importance of global genomic surveillance and data sharing.

## Introduction

In December 2019, the severe acute respiratory syndrome coronavirus 2 (SARS-CoV-2) was first isolated from several workers who were suffering from pneumonia in China (Roychoudhury et al., 2020). It was declared as a global pandemic on 11 March 2020 by the World Health Organization (WHO), which is one of the greatest public health emergencies in human history (Roychoudhury et al., 2020). Globally, the COVID-19 pandemic has resulted in more than 628 million confirmed cases, with over 3.8 million deaths as of 2 November 2022.^1^ The spike (S) protein, this virus’s most important structural protein, plays a dominant role in the viral invasion (Lan et al., 2020; Jackson et al., 2022). The process of SARS-CoV-2 entry is initiated by the interaction between the receptor-binding domain (RBD) in the C-terminus of the S protein sub-segment S1 and angiotensin-converting enzyme 2 (ACE2) receptor on the host cell (Roychoudhury et al., 2020, 2021). This is followed by a conformational change of S protein initiated by type 2 transmembrane serine protease (TMPRSS2) on host cell surface, which allows the virus to enter the cell (Roychoudhury et al., 2021). Then, SARS-CoV-2 hijacks the cellular machinery for viral replication. As the virus continued to evolve, many mutations, especially those in the *S* gene, have gradually increased over time, leading to the emergence of several SARS-CoV-2 variants, and expanding our understanding of the impact of virus variants (WHO, 2022). Viral variants with mutations in the receptor-binding domain (RBD) within the *S* gene that can enhance viral transmissibility and virulence or affect vaccine effectiveness (immune escape potential) and diagnostic testing are designated as variants of concerns (VOCs) by the World Health Organization (WHO; Kumar et al., 2022). Thus far, SARS-CoV-2 has evolved into four previously circulating VOCs (Alpha, Beta, Delta, and Gamma) as well as one currently circulating VOC (Omicron) that has rapidly spread throughout the world (Shrestha et al., 2022; Wang et al., 2022).

The ongoing Omicron (B.1.1.529) variant was first detected in South Africa in mid-November 2021 (Araf et al., 2022; Saxena et al., 2022), and categorized as a VOC subsequently by WHO (Maxmen, 2022). It quickly spread to 80 countries within 3 weeks (Viana et al., 2022). Currently, SARS-CoV-2 Omicron is still the dominant variant circulating worldwide (in more than 170 countries and territories), accounting for >98% of SARS-CoV-2 genomes uploaded to the GISAID database since February 2022 (WHO, 2022). The Omicron variants have continued to evolve, resulting in Omicron subvariants that have been classified into several lineages, namely BA.1, BA.2, BA.3, BA.4, BA.5, and descendent lineages (Shrestha et al., 2022). The first three lineages (BA.1, BA.2, and BA.3) were identified almost simultaneously, and they are different not only from each other but also from previously circulating VOCs (Berkhout and Herrera-Carrillo, 2022; Shrestha et al., 2022). More recently, two additional lineages (BA.4 and BA.5) were first discovered in South Africa and have subsequently spread worldwide (Tegally et al., 2021; Maxmen, 2022). The most recent common ancestor of BA.4 and BA.5 has been estimated to appear in mid-November 2021 (Tegally et al., 2022b).

Omicron is the most divergent variant so far, due to its unprecedented set of non-synonymous mutation sites including 60 substitutions, deletions, and insertions (Kumar et al., 2022). Eight substitutions (K856R, L2084I, A2710T, T3255I, P3395H, and I3758V for ORF1a, and P314L and I1566V for ORF1b) and two deletions (four amino acids) have been identified in ORF1a and ORF1b of the Omicron variant contains (He et al., 2021; Kumar et al., 2022). More than 30 mutations have been identified on S protein of this variant, with half of these mutations (G339D, S371L, S373P, S375F, K417N, N440K, G446S, S477N, T478K, E484A, Q493R, G496S, Q498R, N501Y, and Y505H) accumulated in the RBD, enhancing binding affinity to ACE2 as well as strengthening viral transmission speed (Kumar et al., 2022; Viana et al., 2022). Furthermore, Omicron possesses D614G in the spike protein that shared by five VOCs, correlating with higher viral loads in the upper respiratory tract and a higher rate of infection in younger persons (Korber et al., 2020; Plante et al., 2021). For envelope (E) and membrane (M) proteins, one substitution (T9I) and three substitutions (D3G, Q19E, and A63T) were identified, respectively (He et al., 2021). In addition, three substitutions (P13L, R203K, and G204R) and a deletion of three amino acid residues (ERS31-33del) in the nucleocapsid (N) proteins were also found (He et al., 2021).

Omicron subvariant BA.5.1.3 was first detected in Romania on 24 February 2022, and has since become the world’s prevalent subvariant, identified in 59 countries and territories as of 2 October 2022. The densest concentration of cases has been mainly found in Germany, United States, Denmark, and United Kingdom. Compared with other subvariants, Omicron BA.5.1.3 is more contagious and spreads faster, thanks to the S:V289I mutation (Chen et al., 2021). The S protein of BA.5.1.3 has a common mutation pattern to the BA.4 and BA.5 lineages and is most similar to the BA.2 lineage, excluding 69–70 deletions, L452R, and F486V mutations, and the wild-type amino acid at Q493 (Tegally et al., 2022a).

From 1 August 2022 until early September 2022, a SARS-CoV-2 epidemic raged in Hainan Province in China. In this study, we performed whole-genome SARS-CoV-2 sequencing on RT-PCR positive nasopharyngeal swabs from the city of Sanya (which was the center of the outbreak), detecting the presence of the highly transmissible Omicron BA.5.1.3 subvariant. We then investigated the diversity of 384 genomes including their unique mutations, and compared the binding affinity of several mutations in the S protein. Finally, we conducted phylogenetic analysis and characterized the global distribution of BA.5.1.3.

## Materials and methods

### Sampling

From August 1st to 18th August 2022, as mentioned previously, SARS-CoV-2 positive (RT-PCR detection) oropharyngeal swab samples were preserved in 3 mL viral sample preservation solution (Inactivated) and collected as part of national COVID-19 routine surveillance from the city of Sanya in Hainan Province in China and preserved in viral transport medium (Zhu et al., 2021, 2022). The total RNA of was extracted from 200 μL sample using the QIAamp viral RNA mini kit (Qiagen, Germany) and eluted using 40 μL RNase-free water. The 5 μL eluted water of each sample was conducted to clinical one-step RT-PCR using detection kit for 2019-nCoV (PCR-Fluorescence) according to the manufacturer’s instructions (DaAnGene, China) targeting the *ORF1ab* and *N* genes, respectively. All sampling work was done in collaboration with the national network for COVID-19 surveillance and approved by the Ethics Committee of Sanya People’s Hospital (NO: SYPH-2022-043).

### Whole-genome sequencing of SARS-CoV-2

The DNA of the positive oropharyngeal samples was removed using DNase I, and the concentration of total RNA was determined using Qubit 2.0 (Invitrogen, United States). The sequencing libraries were constructed using a gene-specific multiplexed amplicon-based sequencing strategy (ATOPlex SARS-CoV-2) and sequenced on the MGISEQ-2000 (MGI, China) platform (paired reads 100). The raw reads were qualified using FastQC v0.11.9^2^ (Andrews, 2010), and the clean reads were then aligned to the genome of the Wuhan-Hu-1 virus (MN908947.3) using Bowtie 2 (Langmead and Salzberg, 2012) and assembled by SPAdes v3.15.4 (Bankevich et al., 2012).

### Lineage classification and variant calling

The lineages of whole SARS-CoV-2 genomes were classified using pangoLEARN v4.0^3^ (O’Toole et al., 2021). The global Omicron BA.5.1.3 genomes (sampling location designated as non-China), along with their complete genomes and collection dates, were downloaded from the GISAID database (date of access 2 October 2022).^4^ Those genomes with low coverage and > 0.01% of N were parsed. The mutations of genomes were called using SARS-CoV-2-freebayes with Wuhan-Hu-1 as the reference (Farkas et al., 2021), and the obtained VCF files were filtered and annotated using the SnpEff package (Cingolani et al., 2012). The frequency of each variant was calculated, which was then divided into two groups based on sampling location (non-China or China). The single nucleotide polymorphisms (SNPs) of the amino acids and genomes were summarized and visualized using ESPript v3.0 (Robert and Gouet, 2014) and snipit,^5^ respectively.

### Structural modeling

The native protein structure of hACE2 (1R42) was downloaded from the Protein Data Bank (PDB).^6^ The SARS-CoV-2 RBD structures were modeled using SWISS-MODEL with PDB: 7a91.1 as the template (Waterhouse et al., 2018). Receptor (hACE2)-ligand (RBD) docking was conducted via the Hex protein docking algorithm, using the default parameter settings (shape dimension: 0.6, receptor range: 180, ligand range: 180, distance range: 40, and box size: 10; Macindoe et al., 2010).

### Phylogenetic analysis

Whole genome sequences from this study were aligned using MAFFT v7.471 set to the default parameters (Katoh and Standley, 2013). The alignment was submitted to Nextstrain v12 using SARS-CoV-2 workflow and visualized using auspice (Hadfield et al., 2018). Additionally, the BA.5.1.3 globally-sourced and China-sourced genomes were aligned using MAFFT set to the fast option (FFT-NS-2) and trimmed using Gblocks v0.91b (Castresana, 2000), which were then used to build phylogenetic trees using FastTree v2.1 (Price et al., 2010). The haplotype phylogenetic network of *S* gene was clustered by sampling date and built using PopART v1.7 (Leigh and Bryant, 2015).

### Molecular clock estimation

The genomes from this study and representatives of Nextstrain clades belonging to Omicron were aligned and used to infer its preliminary maximum-likelihood tree, which was also inspected for the presence of a temporal signal using TempEst v.1.5.3 (Rambaut et al., 2016). Bayesian time-calibrated tree was build using BEAST v1.10.4 (Suchard et al., 2018), with a strict molecular clock model, an exponential growth coalescent model, and an HKY + I substitution model (Tegally et al., 2021). The length of the Markov chain of the Monte Carlo (MCMC) tree was 1 × 10^8^, with sampling every 1,000 steps. The result was evaluated using Tracer v1.7.1 (effective sample size >200) and summarized in TreeAnnotator v1.10.4, discarding 10% as burn-in (Rambaut et al., 2018; Suchard et al., 2018).

### Phylogeographic analysis

To infer the phylogenetic diffusion, the genomic alignment and corresponding location traits were jointly inferred at 1 × 10^8^ generations and sampled every 10,000 steps using BEAST v1.10.4 (Suchard et al., 2018). The geographic transition routes (country level) and posterior probability (PP) were summarized and visualized using SPREAD3 v0.9.7.1 with the Bayesian stochastic search variable selection (BSSVS) method (Bielejec et al., 2016).

### Statistical analysis

Statistical plots were conducted using Origin Pro 2021 version. The significant difference was estimated by GraphPad Prism v8.3.0, using either the Mann–Whitney test or Kruskal–Wallis test with *p* < 0.05 indicating statistical significance.

## Results

### Outbreak investigation

**Figure 1:**
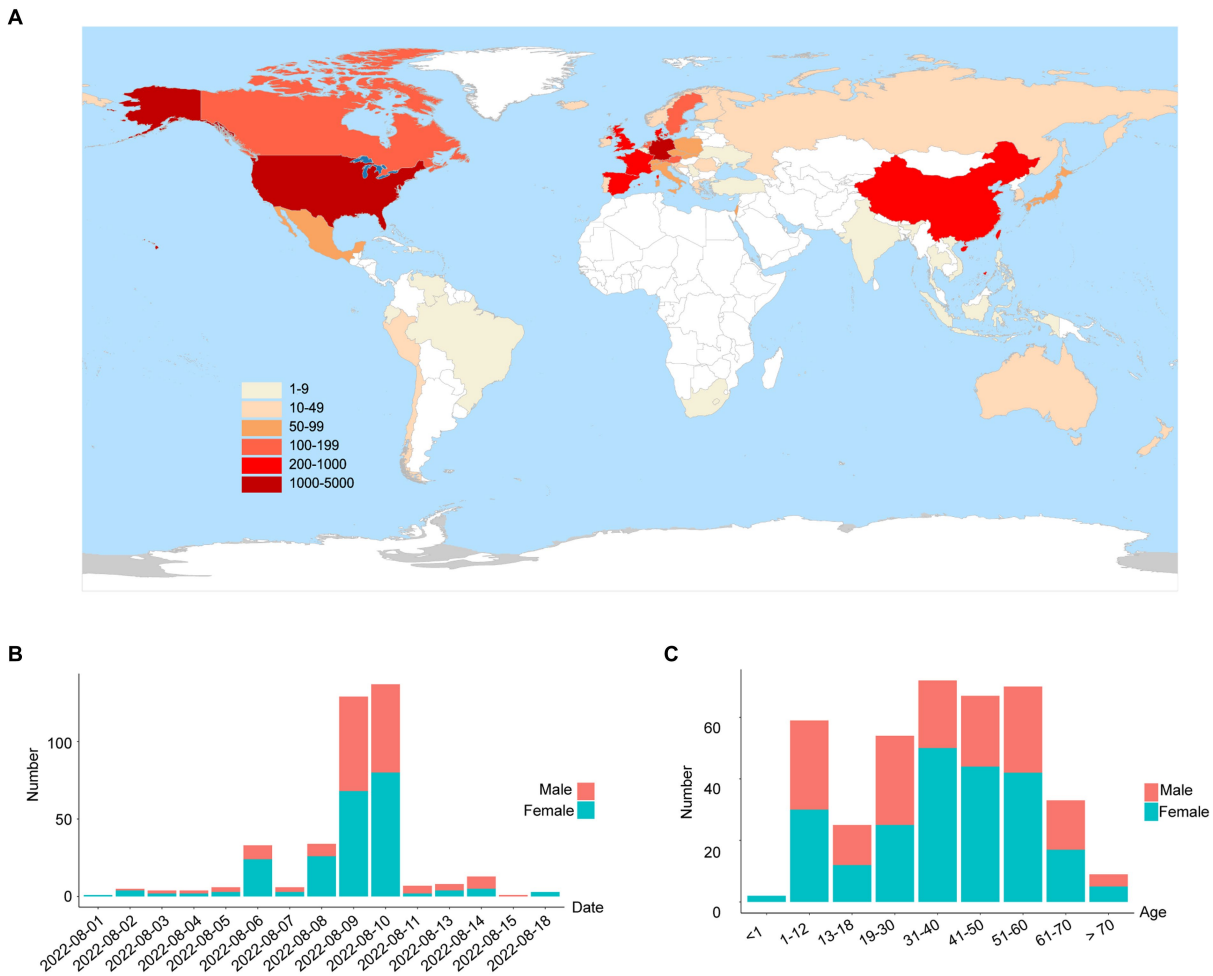
The global emergence of Omicron BA.5.1.3 and cases identified in this study. **(A)** Map showing the global distribution of Omicron BA.5.1.3. The locations of genomes from this study and GISAID (date of access 2 October 2022) were grouped by countries and territories. The shade of each color represents the corresponding confirmed cases per country. **(B)** The sampling date of confirmed cases in the city of Sanya. **(C)** The age range of confirmed cases in Sanya. Orange represents male cases and blue represents female cases.

**Figure 2:**
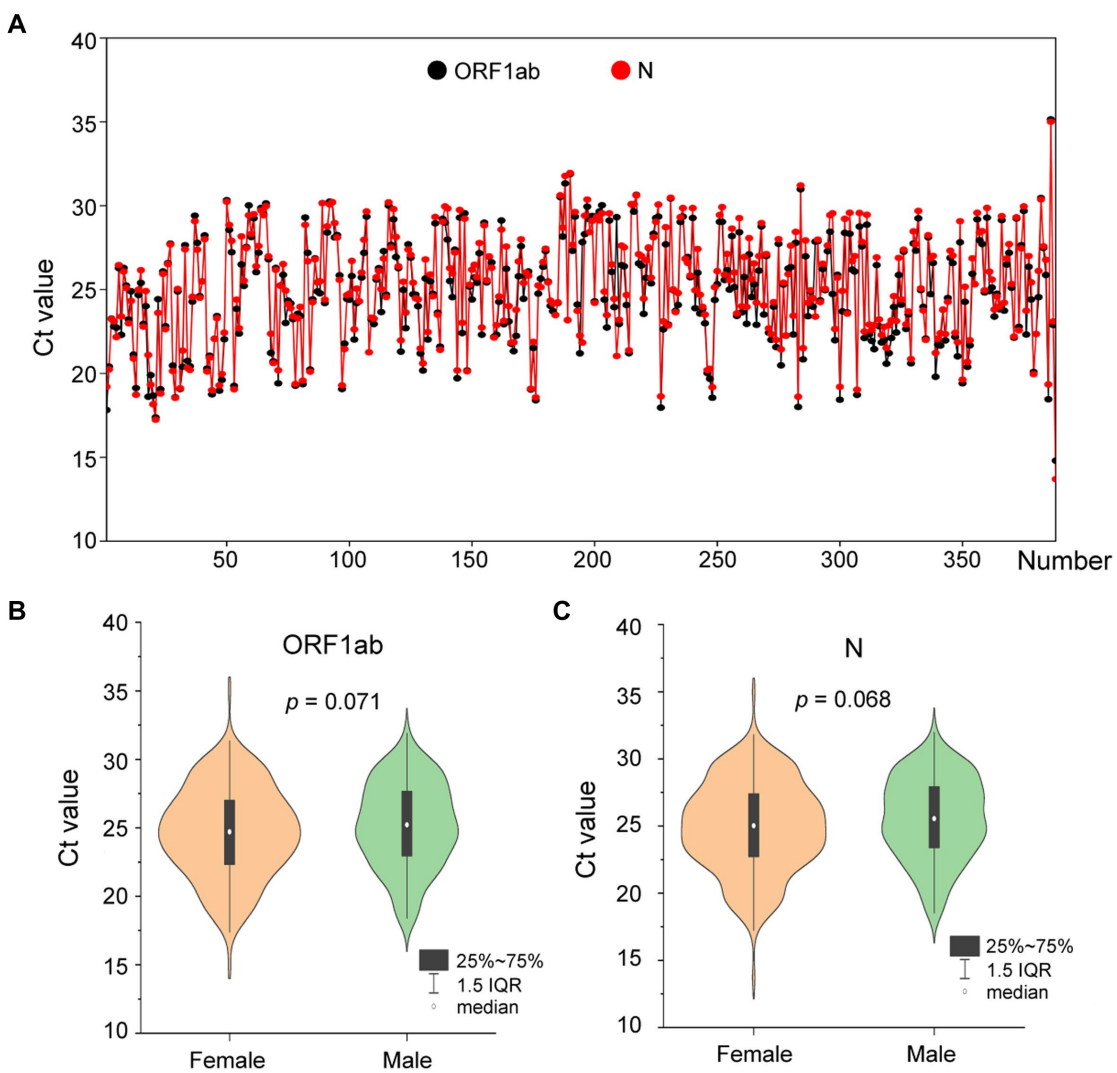
The Ct values of confirmed cases from the city of Sanya. **(A)** The Ct value of each case. The values of *ORF1ab* and *N* genes are labeled with black- and red-filled circles, respectively. **(B)** The comparison between female and male cases based on Ct value of ORF1ab. **(C)** The comparison between female and male cases based on Ct value of N.

On 1 August 2022, a SARS-CoV-2 outbreak erupted in Sanya, the southernmost city on the island province of Hainan in China. This epidemic lasted until early September 2022, resulting in 8,630 confirmed cases with 6,595 cases from the city of Sanya (Supplementary Figure S1). The nasopharyngeal swab specimen of the first case was analyzed using next-generation sequencing, indicating that the causative agent was SARS-CoV-2 Omicron BA.5.1.3 (Nextstrain clade 22B), which had spread to more than 60 countries and territories worldwide at the time this sample was collected (Figure 1A). The outbreak in Sanya has been attributed to China’s first cases of the Omicron BA. 5.1.3. From 1 August 1 to 18 August, a total of 391 SARS-CoV-2 positive specimens with corresponding metadata were randomly collected to perform further analysis (Figure 1B). More than half of the confirmed cases were female (58%, 227/391; Figure 1B). The age of individuals ranged from 2 months to 83.0 years old, with a median of 37.0 (IQR 23.0–53.0) years old (Figure 1C). The cycle threshold (Ct) value of *ORF1ab* gene ranged from 14.8 to 35.2 with median of 24.9 (IQR 22.6–27.3; Figures 2A,B). The Ct value of *N* gene ranged from 13.7 to 35.0, with a median of 25.2 (IQR 22.9–27.6; Figures 2A,C). The difference in Ct values between female and male cases was insignificant (*p* > 0.05) for both *ORF1ab* and *N* gene (Figures 2B,C).

### Evolutionary analysis

**Figure 3:**
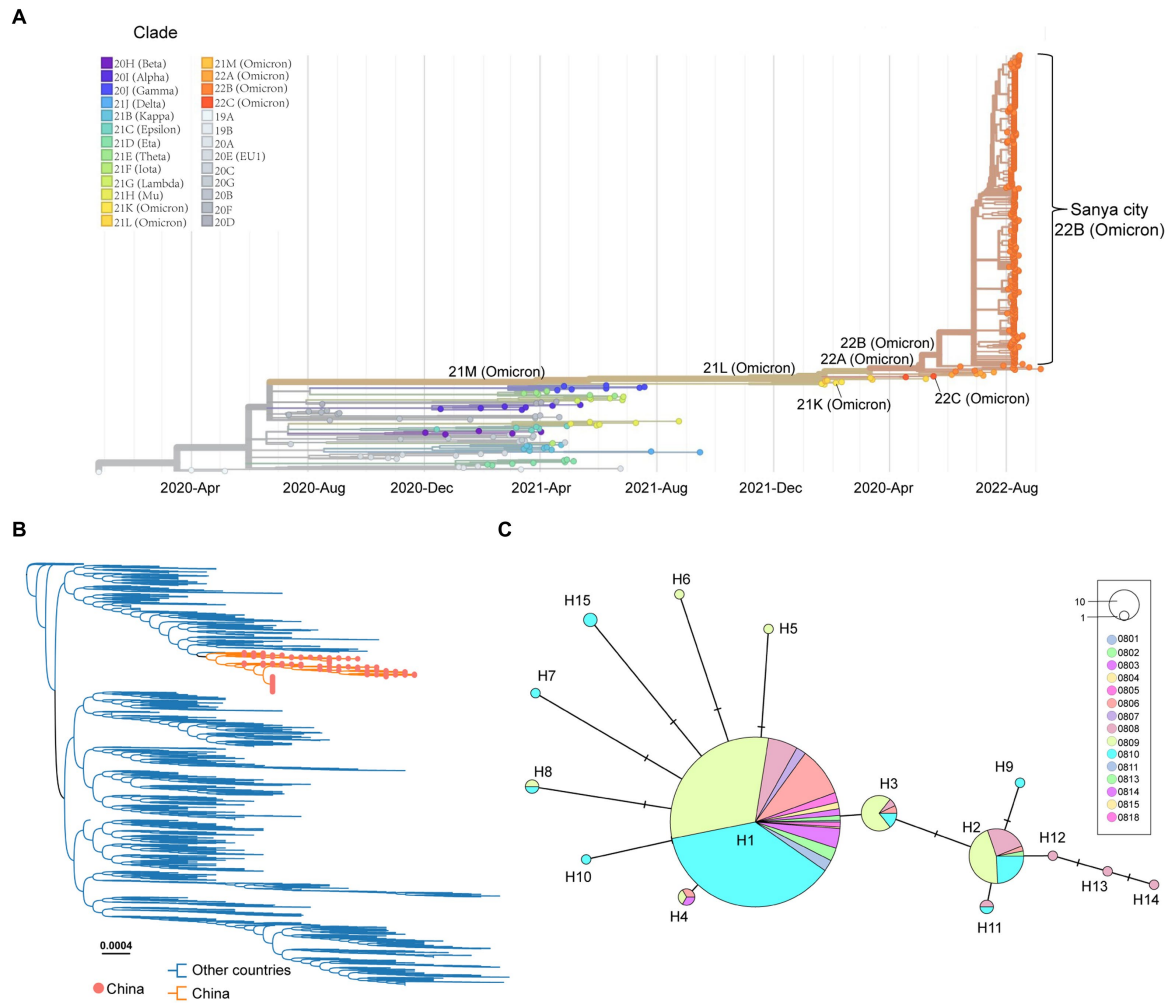
The overview of phylogenetic distribution of genomes identified from the city of Sanya. **(A)** Nextstrain’s phylodynamic analysis of genomes from Sanya City. **(B)** The phylogenetic analysis, including BA.5.1.3genomes from the GISAID database and the city of Sanya. The sequences from this study are labeled with orange-filled circles and orange branches. **(C)** The haplotype network of the *S* genes identified in the city of Sanya. The network is built by PopART using the median-joining method. The size of the circles represents the number of corresponding haplotypes. The color indicates the corresponding sampling date.

After removing the low-quality genomes from the total genomes collected, a total of 384 SARS-CoV-2 genomes were retained for phylogenetic analysis. The results from Nextstrain and Nextclade analyses showed that all these SARS-CoV-2 genomes clustered together and belonged to Omicron BA.5.1.3 (Nextstrain clade 22B; Figure 3A). To assess their detailed phylogenetic relationship, all available BA.5.1.3 Omicron genomes (4,123 genomes retained after parsing) were retrieved from the GISAID database (2 October 2022) and used to build a maximum-likelihood tree. Results revealed that SARS-CoV-2 genomes from the city of Sanya formed a monophyletic cluster within all BA.5.1.3 genomes (Figure 3B). The *S* gene of Omicron BA.5.1.3 genomes from Sanya could be divided into 15 representative haplotypes, which indicated that the vast majority of genomes (83.3%, 320/384) belonged to haplotype 1 (H1), followed by H2 (8.6%, 33/384) and H3 (3.6%, 14/384; Figure 3C).

### Mutational profile

**Figure 4:**
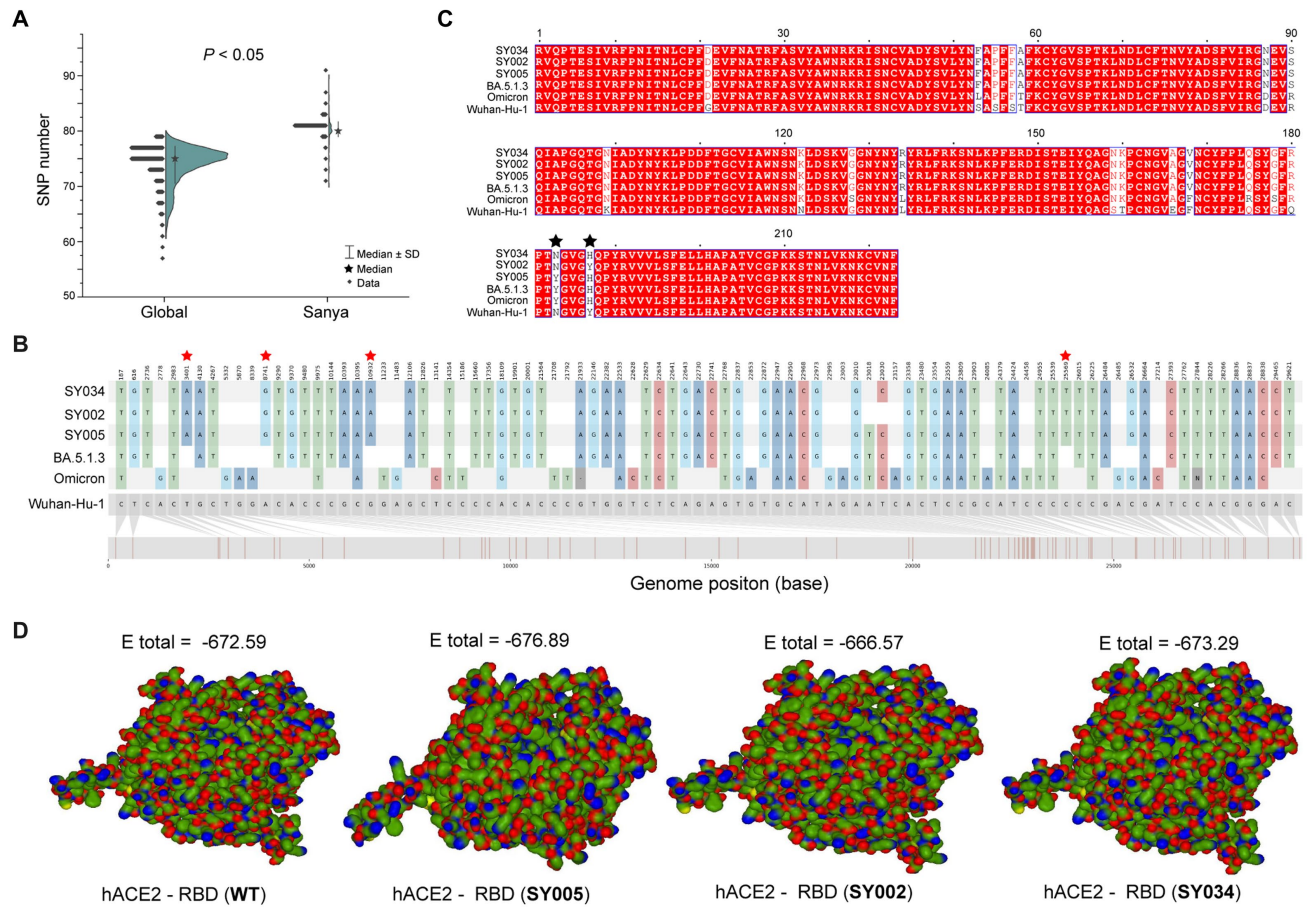
The mutation analyses of whole genomes identified in this study. **(A)** The comparison of SNP numbers of BA.5.1.3 between China (city of Sanya) and non-China (global) genomes. **(B)** The whole genome SNP map with Wuhan-Hu-1, Omicron, BA.5.1.3, SY005, SY002, and SY034. The red stars represent the position of unique mutations. **(C)** The comparison based on amino acid sequences of the RDB. The black stars represent the 501 and 505 positions of S proteins. **(D)** The receptor-ligand docking between hACE2 and the corresponding RBD. The E total value above each structure indicates the docking scores. The Wuhan-Hu-1, Omicron, and BA.5.1.3 represent the genomes of the original SARS-CoV-2 (MN908947.3), the first Omicron variant (EPI_ISL_6640917), and the first BA.5.1.3 variant (EPI_ISL_14026136), which are available in public databases, respectively. WT here stands for Wuhan-Hu-1.

In total, the genomes from China (in the city of Sanya) had marked hypermutation, including 70 to 90 mutations (containing deletions) with a median of 80 (IQR 80–81), showing a significant difference (*p* < 0.05) with non-China-sourced genomes (global; Figure 4A). During the outbreak, 25 core mutations (frequency > 98%) were identified in ORF1ab (Supplementary Table S1), which also included three unique mutations (Y1064N, S2844G, and R3574K) that were exclusive or non-core to global BA.5.1.3 genomes (Table 1; Figure 4B).

**Table 1:** Unique mutations identified in BA.5.1.3 genomes from China.

Gene	Protein	Position (aa)	Mutation (nt)	Mutation (aa)	Frequency	Percentage (%)	Synonymous mutation
ORF1ab	nsp3	1,064	T3190A	Y1064N	384	100	N
ORF1ab	nsp4	2,844	A8530G	S2844G	384	100	N
ORF1ab	nsp6	3,574	G10721A	R3574K	384	100	N
ORF3a	-	74	C222T	S74S	384	100	Y

nsp, non-structural protein; N, No; Y, Yes.

Obviously, the most mutations were located in the *S* gene, including 35 core mutations (frequency > 99% except for N501Y and Y505H) with one synonymous mutation (D1146D/C3438T). Half (17) of these core mutations of dominating genomes (H1) from the city of Sanya were located in the RBD domain (Figure 4C). The mutation profile of the *S* gene shared the core mutations of global Omicron BA.5.1.3 genomes (Table 2). In addition, all Sanya-sourced and globally sourced BA.5.1.3 genomes (>99.5%) shared the V289I mutation that was absent in the core mutations of any Omicron sub-lineages, while BA.5.1.3 did not contain the G142D mutation that shared by all other Omicron variants (BA.1, BA.2, BA.4, BA.5, BA.2.12.1, BQ.1, and XBB; Hodcroft, 2021). Interestingly, two revertant mutations with a relatively low frequency were found in position 501 (13.8%, 53/384) and position 505 (10.1%, 39/384), both belonging to the RBD domain (Table 2; Figure 4C).

**Table 2:** Mutations found in S protein of BA.5.1.3 genomes from China.

Position (aa)	Mutation (nt)	Mutation (aa)	Frequency	Percentage of China (%)	Percentage of the world (%)	Synonymous mutation
19	C56T	T19I	381	99.2	97.0	N
24	deletion	deletion	382	99.5	97.2	N
25	deletion	deletion	382	99.5	97.2	N
26	deletion	deletion	382	99.5	97.2	N
27	deletion	deletion	382	99.5	97.2	N
69	deletion	deletion	380	99.0	97.0	N
70	deletion	deletion	380	99.0	97.0	N
213	T638G	V213G	384	100	99.3	N
289	G865A	V289I	384	100	99.2	N
339	G1016A	G339D	384	100	91.3	N
371	C1112T	S371F	384	100	87.8	N
373	T1117C	S373P	384	100	91.6	N
375	deletion	S375F	384	100	91.3	N
376	deletion	T376A	384	100	91.3	N
405	G1213A	D405N	384	100	92.4	N
408	A1224C	R408S	384	100	89.6	N
417	G1251T	K417N	384	100	91.1	N
440	T1320G	N440K	384	100	85.1	N
452	T1355G	L452R	384	100	92.3	N
477	G1430A	S477N	384	100	92.7	N
478	C1433A	T478K	384	100	92.7	N
484	A1451C	E484A	384	100	93.0	N
486	T1456G	F486V	384	100	93.1	N
498	A1493G	Q498R	384	100	91.6	N
501	A1501T	N501Y	331	86.2	92.4	N
505	T1513C	Y505H	345	89.8	92.8	N
614	A1841G	D614G	384	100	99.8	N
655	C1963T	H655Y	384	100	99.9	N
679	T2037G	N679K	384	100	99.3	N
681	C2042A	P681H	384	100	99.2	N
764	C2292A	N764K	384	100	95.8	N
796	G2386T	D796Y	384	100	99.4	N
954	A2862T	Q954H	384	100	99.5	N
969	T2907A	N969K	384	100	99.6	N
1,146	C3438T	D1146D	381	99.2	97.7	Y

N, No; Y, Yes.

We detected three (two synonymous and one non-synonymous) core (100%) mutations in ORF3a (T64T, S74S, and T223I), including one novel synonymous mutation (S74S/C222T; Table 1; Figure 4B). Additionally, 13 mutations, namely one non-synonymous mutation (T9I) in E, two non-synonymous mutations (D3N and Q19E) in M, one synonymous mutation (C15C/T45C) in ORF7a, one synonymous mutation (L18L) in ORF7b, seven non-synonymous mutations (P13L, ERS31-33del, RG203KR, and S413R) in N, and one non-synonymous mutation (L37F) in ORF10, were identified as core mutations. Meanwhile, four core mutations were identified in the non-coding regions, including C241T (5′UTR), C27889T (between ORF7b and ORF8), A28271T (between ORF8 and N), and 29734_29759del (3′UTR).

### Receptor-ligand docking

The binding affinity between SARS-CoV-2 RBD and hACE2 accounted for variant infectivity and differed between variants (Kumar et al., 2022). Based on the haplotype analysis, the genome sequences of SY005, SY002, and SY034 were designated as representatives of H1, H2, and H3, respectively. We compared the docking energies of the RBD of H1 (SY005, EPI_ISL_15940718), H2 (SY002, EPI_ISL_15940715), and H3 (SY034, EPI_ISL_15940747) binding to hACE2 (Figure 4D), respectively. Results revealed that the docking energy of Wuhan-Hu-1 with hACE2 was −672.59, which was lower than that of the dominant strain SY005 (−676.89), indicating the higher infectivity and transmissibility of the virus in Sanya. The revertant mutation in position 501 (SY034) had a slightly lower docking energy (−673.29) compared with SY005, signifying that N501Y in the RBD promoted viral binding (Figure 4D). The two mutations in positions 501 and 505 reverted simultaneously (SY002) and showed more lower docking energy compared with SY005 and SY034, indicating that both N501Y and Y505H in the RBD might promote viral infectivity (Figure 4D).

### Molecular clock analysis

**Figure 5:**
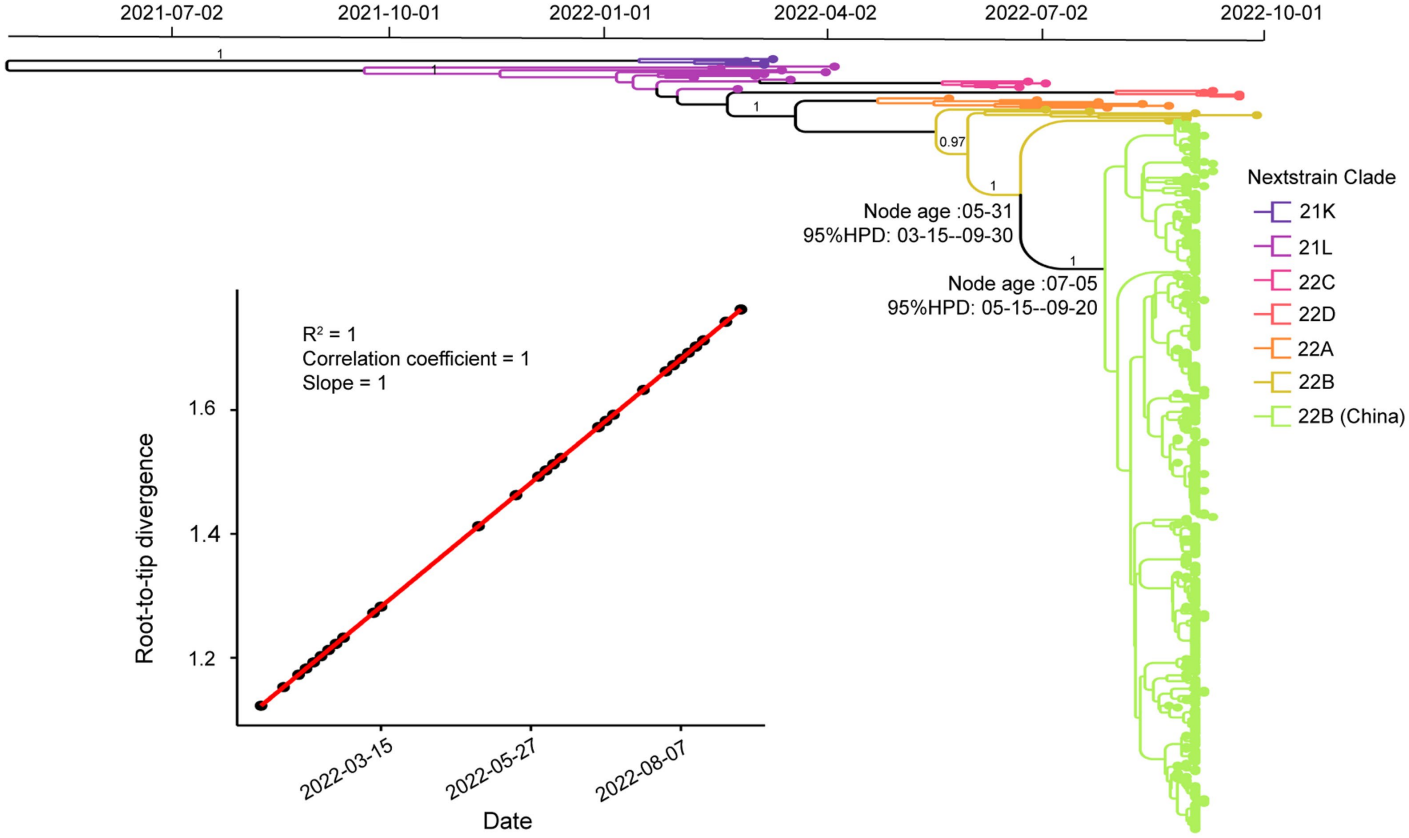
Temporal analysis of Omicron BA.5.1.3 genomes from China. The branches are labeled with different colors according to Nextstrain’s clades. The posterior probabilities are indicated on corresponding branches. The tMRCA of genomes from Sanya is labeled on their corresponding nodes with 95% highest probability density (HPD) values. The molecular clock signal plot revealed the correlation between sampling dates and the root-to-tip distance. The tMRCA was based on collection dates.

To investigate the phylogenetic history of BA.5.1.3 genomes from Sanya, the representatives of Omicron genomes were used as background to conduct a molecular clock analysis. The temporal signal test showed that the sampling dates were correlated with the root-to-tip distance, implying a significant (R^2^ = 1) temporal signature (Figure 5). The Bayesian analysis estimated the substitutions of Omicron BA.5.1.3 genomes at 8.896 × 10^−4^ nucleotide changes per site per year, which was a reliable rate compared to other lineages (Tegally et al., 2021). The time-calibrated phylogenetic tree tracked that the most recent common ancestor (tMRCA) of all BA.5.1.3 genomes from Sanya emerged in early July 2022 with 95% highest posterior density (HPD) ranging from the middle of May to the middle of September 2022 (Figure 5).

### Spatial dynamics

To illustrate the population genetics, the Bayesian skyline analysis was conducted based on global BA.5.1.3 genomes, including those from SARS-CoV-2 Omicron BA.5.1.3 outbreak in Sanya. The result showed that the population size of Omicron BA.5.1.3 grew steadily since its emergence in February 2022 and reached the relatively peak in mid-July 2022 (Supplementary Figure S2). The population size reached the summit at the end of August 2022 and decreased starting from that time (Supplementary Figure S2). To infer their spatial spread, the Omicron BA.5.1.3 genomes were first split into different groups based on their corresponding countries and territories. We performed Bayesian phylogeographic analysis according to the BSSVS method, detecting the presence of 18 migration links in the transmission processes of BA.5.1.3 worldwide, with posterior probability (PP) values of 1 (Supplementary Figure S3). Obviously, certain European and European-adjacent countries, especially Germany and United Kingdom, may have played a pivotal role in BA.5.1.3 transmission (Supplementary Figure S3; Supplementary Table S2).

## Discussion

The failure to control the transmission of SARS-CoV-2 in many countries and territories around the world has led to the development of hypotheses suggesting that substantial evolutionary mutations accounting for the emergence of virus variants, particularly the Omicron (Fontanet et al., 2021; Mascola et al., 2021; Tegally et al., 2021). In this study, we investigated the China’s first SARS-CoV-2 Omicron BA.5.1.3 outbreak, which has caused more than 8,500 confirmed cases and led to the lockdown of the tropical island known as Hainan Province.

The number of confirmed cases rose quickly in the initial stages of this outbreak. More than half these cases are female (58%), differing from those of the WT (<50%), Alpha (39%), Beta (27%), and Delta (49%) variants (Huang et al., 2020; Ong et al., 2022). In this cohort, we also unexpectedly observed three infants (≤1 years old) and 11 persons of advanced age (≥70 years old), suggesting that people of all ages may be susceptible to Omicron BA.5.1.3. The viral loads in the upper respiratory tract caused by BA.5.1.3 among different age and sex groups show no obvious difference. Surprisingly, the BA.5.1.3 in this study seems to result in higher overall viral load (with a median Ct value of 25.2 for *N* gene from here) compared with BA.1 (median = 26.29) and BA.2 (median = 25.48) in symptomatic respondents (Whitaker et al., 2022). Meanwhile, the overall median Ct value of 26.4 from large-community surveillance study (Walker et al., 2021) indicated potentially faster transmission and enhanced viral fitness of BA.5.1.3.

The highly mutated SRAS-CoV-2 Omicron BA.5.1.3 have accumulated 35 core mutations in the S protein, higher than that found in the S proteins of Alpha (10), Beta (11), Delta (12), Gamma (9), and Omicron BA.2 (31), though lower than that found in the S protein of Omicron BA.1 (36; Elbe and Buckland-Merrett, 2017). Furthermore, this finding identically reflected the mutational pattern of BA.5.1.3 as it circulated around the world. Half of these mutations are located in the RBD, resulting in higher binding affinity and selective advantage. The receptor-ligand docking estimation confirmed that N501Y and Y505H mutations of the S protein in SARS-CoV-2 enhance its binding affinity to hACE2, suggesting a higher rate of potential immune escape (Tian et al., 2021; Kumar et al., 2022). The S:V289I mutation is detected in all genomes from Sanya and > 99.5% BA.5.1.3 genomes from the GISAID database. Globally, genomes containing the S:V289I mutation accounted for 0.36% of all SARS-CoV-2 genomes, primarily dominated and prevalent in AY.47 (73.55%) and BA.5.1.3 (20.73%; Elbe and Buckland-Merrett, 2017; Chen et al., 2021). The S:G142D mutation is in about half (48.96%) of all SARS-CoV-2 genomes, mainly in BA.2 (18.00%), AY.4 (9.35%), BA.2.12.1 (4.08%), BA.5.2.1 (3.76%), BA.5.2 (3.37%), AY.103 (3.11%), BA.2.9 (3.11%), and BA.5.1 (3.05%; 2 October 2022; Chen et al., 2021). Thus, the absence of S:G142D mutation and presence of S:V289I mutation could be potential markers of BA.5.1.3 sub-lineage when compared with other sub-lineages (Chen et al., 2021; Hodcroft, 2021).

Three (Y1064N, S2844G, and R3574K) unique core (100%) mutations in ORF1ab are identified in BA.5.1.3 genomes from this study. Before 1 August 2022, there are relatively few genomes (94 belonging to 30 sub-lineages) harboring the ORF1a:Y1064N mutation, with only 5 genomes in BA.5.1.3, and 2,754 genomes (belonging to 46 sub-lineages) possessing the ORF1a:R3574K mutation with only 670 genomes in BA.5.1.3 from public database (Chen et al., 2021). However, the ORF1a:S2844G has not so far been detected in BA.5.13 genomes. Given that other core mutations are identical to publicly available BA.5.1.3 genomes, these unique core mutations of genomes from Sanya indicate that the Omicron BA.5.1.3 outbreak shares the same transmission chain. In addition, its three unique core mutations are within non-structural protein (nsp) 3, nsp4, and nsp6, respectively, which play different but equally essential roles in the viral life cycle (Wu et al., 2020; Armstrong et al., 2021; Wang and Cheng, 2022). As the largest non-structural protein, nsp3 plays a pivotal role in SARS-CoV-2 replication and transcription complexes assembly (Armstrong et al., 2021). When nsp4 binds with nsp3, this can cause membrane rearrangement and generate double-membrane vesicles with nsp6 protein as the connectors (Sakai et al., 2017; Ricciardi et al., 2022). Thus, the three unique core mutations may enhance the interaction of these non-structural proteins, resulting in more efficient viral replication.

The time-resolved maximum clade credibility (MCC) analysis estimated that the emergence of tMRCA was 1 month before the Hainan Province outbreak. Due to the earlier use of regular and large-scale nucleic acid amplification tests earlier in China, it seems impossible that the virus could circulate in Sanya City for so long without being detected, suggesting that this Omicron BA.5.1.3 strain might have evolved and adapted for some time before spreading to China. However, the detailed transmission routine of the Omicron BA.5.1.3 to China is still unclear.

This study also has some limitations. First, the clinical symptoms were unavailable to investigate the symptomatic differences from other lineages. Second, the nasopharyngeal swab specimens were only collected once, thus limiting us from monitoring long-term viral load changes. In addition, we were unable to sequence the whole genomes from all positive specimens, indicating of a potential underestimation of population size and mutations.

## Conclusion

Our study tracks the BA.5.1.3 outbreak in the city of Sanya in Hainan Province in China, delineates its mutational profile (including unique mutation), and estimates the timing of its emergence. Our results highlight the importance of genomic surveillance and of carrying out collaboration and sharing of genomic data worldwide for an effective response to the COVID-19 pandemic.

## Data availability statement

The datasets presented in this study have been deposited in the GISAID database (https://www.gisaid.org/) under accession numbers EPI_ISL_15940714 to EPI_ISL_15941103, and EPI_ISL_15942295.

## Author contributions

WZ and CW conceived and designed this study. XW, XZ, YL, and LH collected samples and conducted the experiments. WZ analyzed the data and prepared all the figures. WZ and XW wrote the first manuscript. WZ, CW, and JY revised the manuscript. All authors contributed to the article and approved the submitted version.

## Funding

This study was supported by Golden Seed Program of Beijing Chaoyang Hospital (CYJZ202220) and also supported by the innovation platform for Jianguo Xu Academicians of Hainan Province.

## Conflict of interest

The authors declare that the research was conducted in the absence of any commercial or financial relationships that could be construed as a potential conflict of interest.

## Publisher’s note

All claims expressed in this article are solely those of the authors and do not necessarily represent those of their affiliated organizations, or those of the publisher, the editors and the reviewers. Any product that may be evaluated in this article, or claim that may be made by its manufacturer, is not guaranteed or endorsed by the publisher.

## Footnotes

^1^https://covid19.who.int/

^2^https://github.com/s-andrews/FastQC

^3^https://github.com/cov-lineages/pangoLEARN

^4^https://gisaid.org/

^5^https://github.com/aineniamh/snipit

^6^https://www.rcsb.org/
