# Supplementary material for: Tracking the first SARS-CoV-2 Omicron BA.5.1.3 outbreak in China
Source: Front Microbiol. 2023 May 18;14:1183633. doi: 10.3389/fmicb.2023.1183633 (PMC10232789; doi:10.3389/fmicb.2023.1183633)
Supplement: Supplementary file 1 [file Table_1.DOCX]

**Tracking the First SARS-CoV-2 Omicron BA.5.1.3 Outbreak in China**

Table S1 Mutations found in ORF1ab

| Position (aa) | Mutation (nt) | Mutation (aa) | Frequency | Percentage of China (%) | Synonymous mutation |
| --- | --- | --- | --- | --- | --- |
| 135 | T405G | S135R | 384 | 100 | N |
| 842 | C2525T | T842I | 384 | 100 | N |
| 924 | C2772T | F924F | 384 | 100 | Y |
| **1064** | **T3190A** | **Y1064N** | **384** | **100** | **N** |
| 1307 | G3919A | G1307S | 384 | 100 | N |
| 1352 | C4056T | A1352A | 384 | 100 | Y |
| **2844** | **A8530G** | **S2844G** | **384** | **100** | **N** |
| 3027 | C9079T | L3027F | 384 | 100 | N |
| 3053 | A9159G | V3053V | 377 | 98.2 | Y |
| 3090 | C9269T | T3090I | 384 | 100 | N |
| 3255 | C9764T | T3255I | 384 | 100 | N |
| 3311 | C9933T | D3311D | 384 | 100 | Y |
| 3395 | 10182_10184del | P3395H | 384 | 100 | N |
| **3574** | **G10721A** | **R3574K** | **384** | **100** | **N** |
| 3675 | deletion | deletion | 384 | 100 | N |
| 3676 | deletion | deletion | 384 | 100 | N |
| 3677 | deletion | deletion | 384 | 100 | N |
| 3965 | G11895A | E3965E | 384 | 100 | Y |
| 4205 | C12615T | I4205I | 384 | 100 | Y |
| 4715 | C14144T | P4715L | 384 | 100 | N |
| 5150 | C15450T | L5150L | 384 | 100 | Y |
| 5716 | C17146T | R5716C | 384 | 100 | N |
| 5967 | A17899G | I5967V | 384 | 100 | N |
| 6564 | C19691T | T6564I | 384 | 100 | N |
| 6597 | A19791G | E6597E | 384 | 100 | Y |

The unique mutations are labeled with bold. N = No; Y = Yes.

Table S2 The spread routes of global BA.5.1.3 with supported posterior probabilities and bayes factors (BF)

| Number | From | To | Posterior probability | Bayes factor |
| --- | --- | --- | --- | --- |
| 1 | Austria | Germany | 1 | > 10000 |
| 2 | Belgium | Germany | 1 | > 10000 |
| 3 | Canada | UK | 1 | > 10000 |
| 4 | Chile | UK | 1 | > 10000 |
| 5 | Denmark | Germany | 1 | > 10000 |
| 6 | Denmark | UK | 1 | > 10000 |
| 7 | France | Germany | 1 | > 10000 |
| 8 | Germany | Ireland | 1 | > 10000 |
| 9 | Germany | Italy | 1 | > 10000 |
| 10 | Germany | Japan | 1 | > 10000 |
| 11 | Germany | Peru | 1 | > 10000 |
| 12 | Germany | South Africa | 1 | > 10000 |
| 13 | Germany | Spain | 1 | > 10000 |
| 14 | Germany | US Virgin Islands | 1 | > 10000 |
| 15 | Germany | UK | 1 | > 10000 |
| 16 | Netherlands | UK | 1 | > 10000 |
| 17 | Norway | UK | 1 | > 10000 |
| 18 | Philippines | UK | 1 | > 10000 |


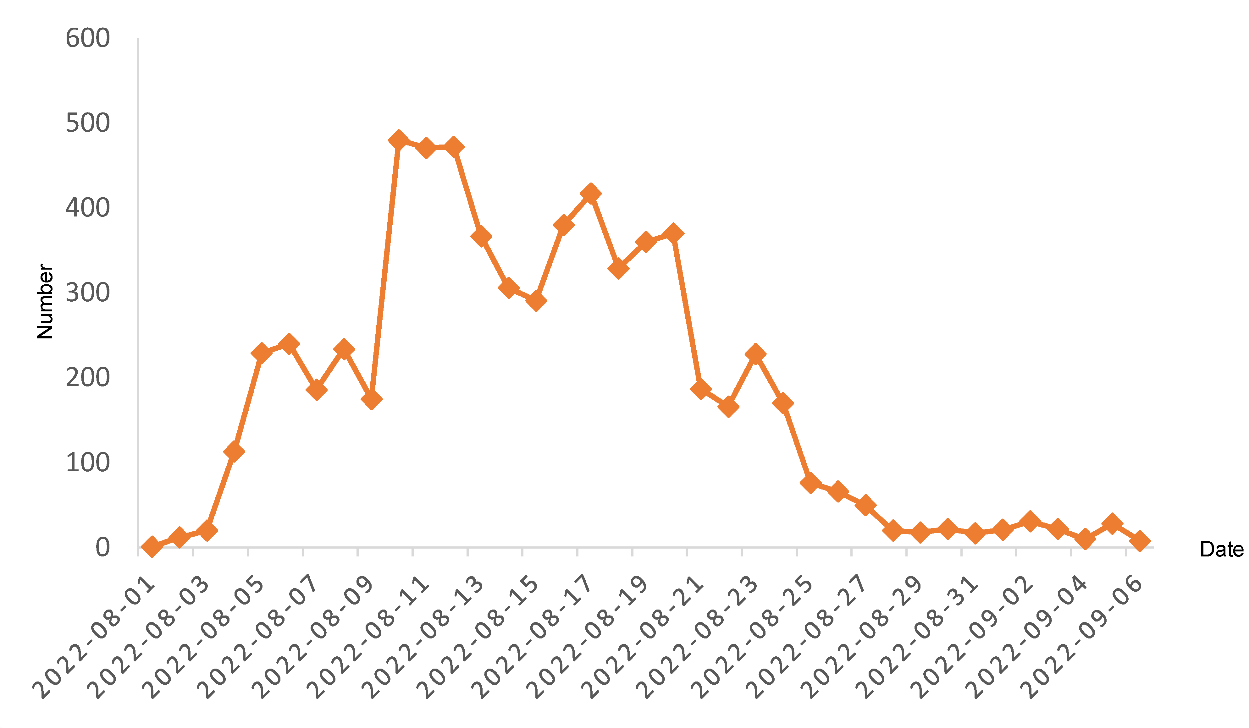


Figure S1 The overall of confirmed cases in each day during this outbreak in Sanya City.


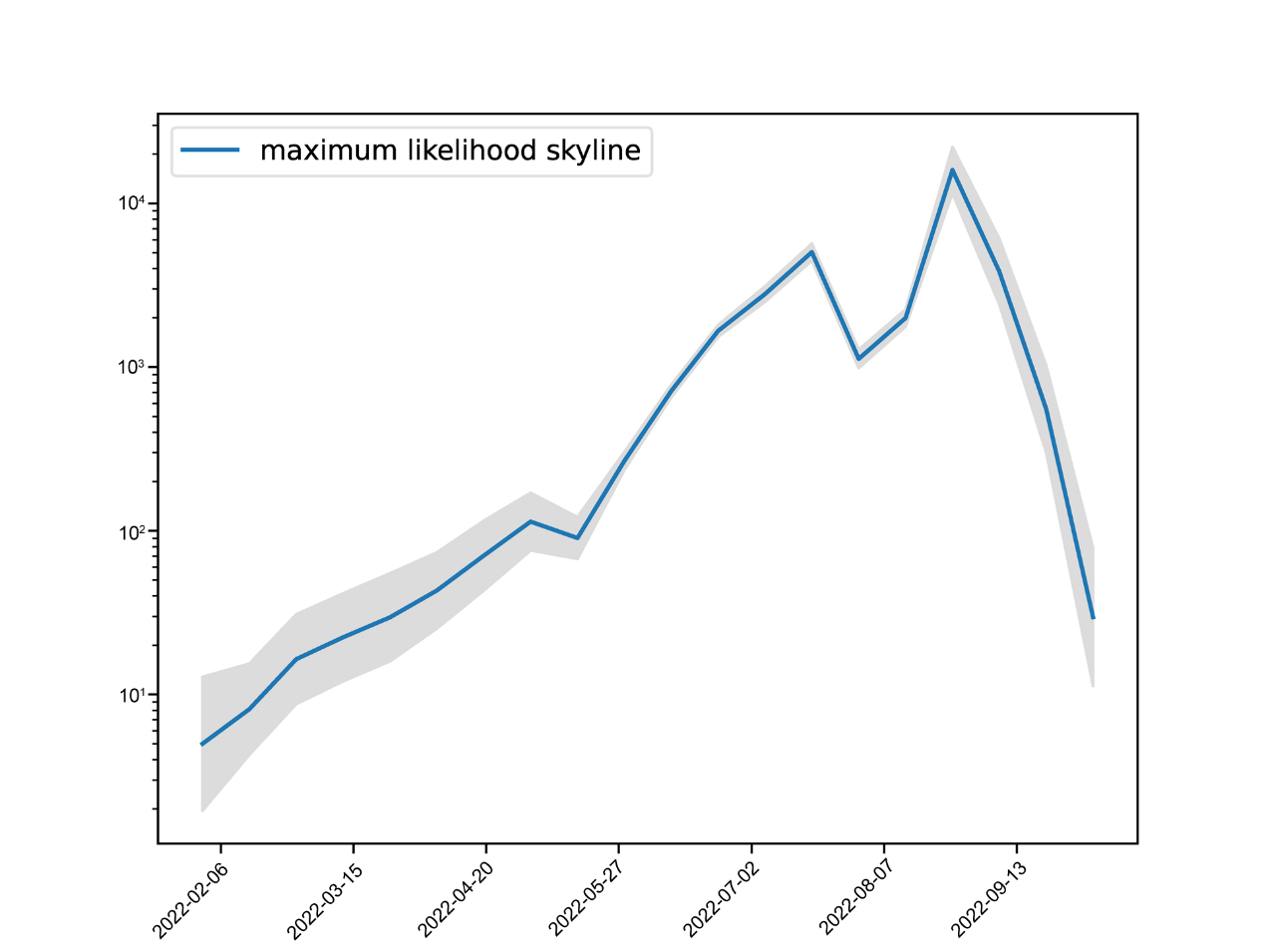


Figure S2 Bayesian skyline plot (BSP) of the complete BA.5.1.3 genomes all over the world. The blue line indicates the mean value of genetic diversity, and the gray blue shading shows the 95% confidence interval.


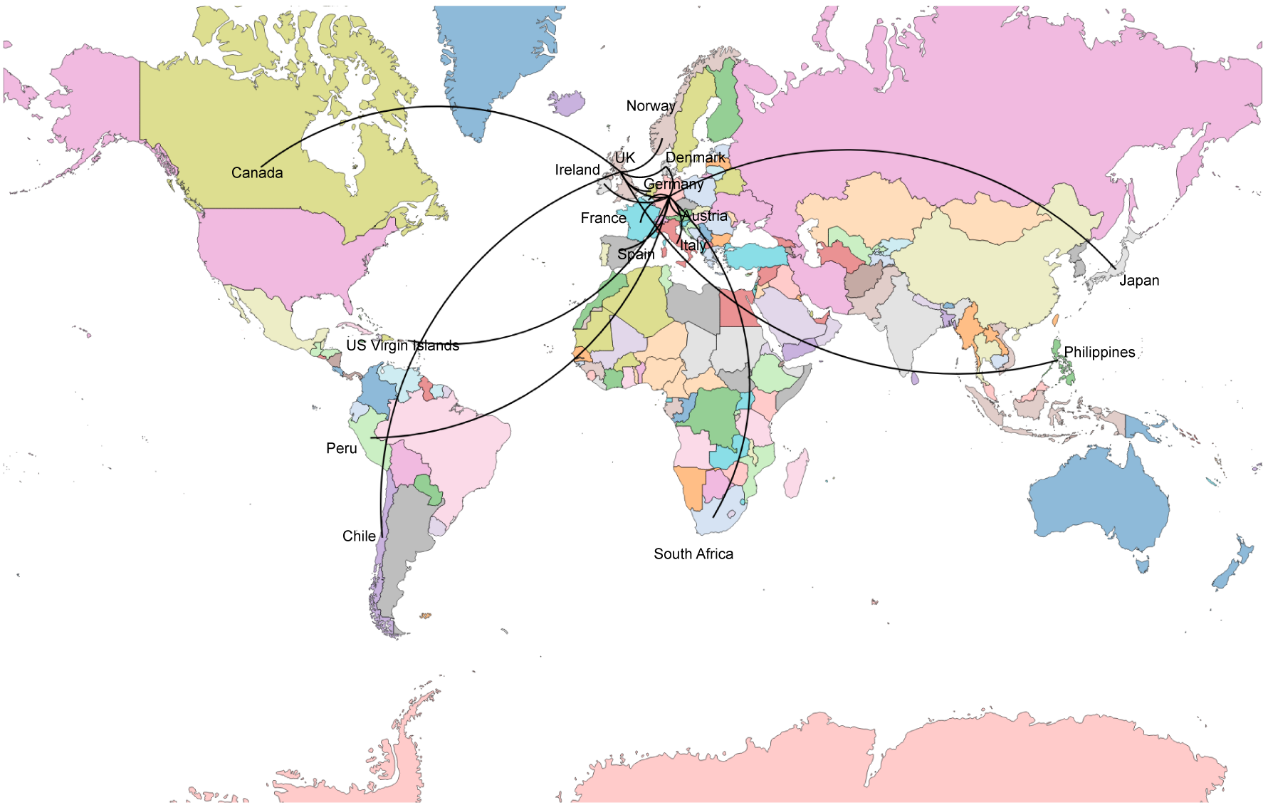


Figure S3 The geographical distribution of Omicron BA.5.1.3 around the world. Only migrations with posterior probability values of 1 are shown. The map is colored according to countries and territories.
